# Supplementary material for: Deletion of wfs1 Impairs Oligodendrocyte Precursor Cells Dorsal Distribution and Myelination Through the wfs1-hmgcs1 Axis in Zebrafish
Source: Biology (Basel). 2026 Aug 16;15(16):1402. doi: 10.3390/biology15161402 (PMC13510151; doi:10.3390/biology15161402)
Supplement: Supplementary file 1 [file biology-15-01402-s001.zip › File S1-Transcriptome sequencing data and differentially expressed genes (DEGs) in wfs1-- zebrafish at 5 dpf/2.KEGG/wfs1KOvsWT/UP/src/dre05168.html]

dre05168


- dre:791704

- Up regulated genes

791704(1.64775824422649)

- dre:100149189

- Up regulated genes

100149189(1.21378518263532)

- dre:58113

- Up regulated genes

58113(1.99980927955512)

- dre:30762

- Up regulated genes

30762(1.51549567324805)
- dre:368878

- Up regulated genes

368878(2.50701188401484)
- dre:30763

- Up regulated genes

30763(5.58822372755117)
- dre:571282

- Up regulated genes

571282(1.90251155561911)

- dre:768248

- Up regulated genes

768248(6.07490717007993)

- dre:561021

- Up regulated genes

561021(2.14284154145004)

- dre:556826

- Up regulated genes

556826\_1(2.25777649220643)

- dre:100330617

- Up regulated genes

100330617\_1(1.54741913113035)

- dre:100000252

- Up regulated genes

100000252\_3(1.94434154854557) 100000252(1.09163460881879)

- dre:564266

- Up regulated genes

564266(2.42311476837666)

- dre:30762

- Up regulated genes

30762(1.51549567324805)
- dre:368878

- Up regulated genes

368878(2.50701188401484)
- dre:30763

- Up regulated genes

30763(5.58822372755117)
- dre:571282

- Up regulated genes

571282(1.90251155561911)

Close
